# A giant ganglion cyst of the semimembranosus tendon: a case report

**DOI:** 10.4076/1757-1626-2-8305

**Published:** 2009-08-05

**Authors:** Sunil Garg, Talal Al-Jabri, Sanjay Mutnal, Farid Moftah

**Affiliations:** 1 Trauma and Orthopaedics, Queen Mary’s Hospital, South London Healthcare NHS TrustFrognal Avenue, Sidcup, DA146LTUK; 2 Trauma and Orthopaedics, Darrent Valley HospitalDartford, DA28DAUK

## Abstract

We report a rare case of a ‘giant ganglion’ with 24 × 10 × 12 cm dimensions originating from the semimembranosus tendon. The patient presented with chronic pain and a palpable mass in his left calf located between the superior aspect of the popliteal fossa and the distal third of the calf. MRI revealed the mass to be a ganglion in close relation to the semimembranosus muscle at its attachment to the tibia. The patient was operated on and had complete resolution of symptoms postoperatively. To the best of our knowledge there are no other case reports in the literature of ganglion cysts of similar size arising from the tendon of semimembranosus. A brief review of the literature is included.

## Introduction

A ganglion is best described as a cyst filled with colloid material met within the vicinity of a joint or tendon sheath. They most commonly occur on the dorsal aspect of the wrist [1]. Popliteal (Baker’s) cysts are commonly reported in patients with underlying osteoarthritis or rheumatoid arthritis and are usually diagnosed clinically but may require the aid of ultrasonagraphy or magnetic resonance imaging (MRI) for further structural detail. The popliteal fossa and calf is an unusual location for a ganglion [2]. Ganglia in the region of the knee are usually situated over the interval between the femur and tibia, most often on the lateral aspect of the joint and may attain the size of a walnut [3]. We report an interesting case where a ‘giant ganglion’ was identified in the popliteal and calf region mimicking an unruptured Baker’s cyst.

## Case precentation

A 50-year-old Caucasian male presented to clinic with a mass in his left popliteal fossa and calf which had been increasing in size over the past six months. This had been associated with intermittent pain in the popliteal fossa. The patient also, felt the mass to be aesthetically displeasing.

**Figure 1.**
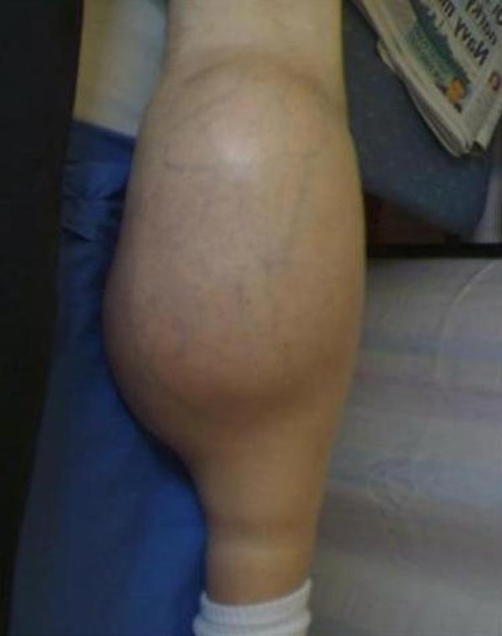
Posterior aspect of the left leg showing the location of the mass.

**Figure 2.**
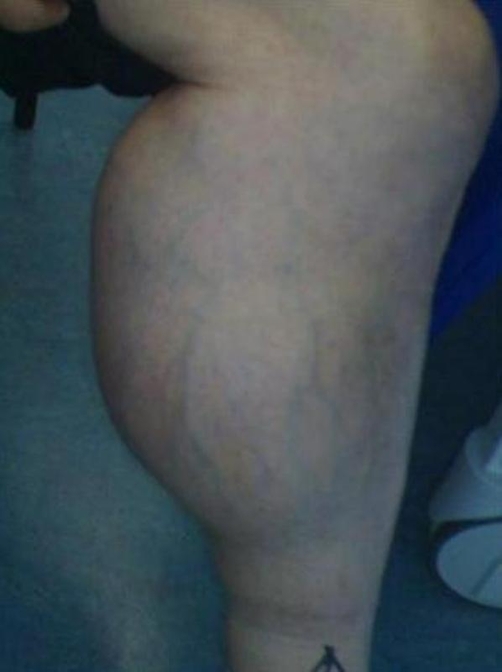
Medial view of the mass in the left leg.

Physical examination revealed a non-tender swelling extending from the superior aspect of the popliteal fossa to the junction of the middle and distal third of the calf. The mass was cystic in consistency and transilluminated with a well defined margin. Flexion at the knee was restricted due to the size of the swelling. The ankle had an uncompromised range of movement (Figures 1 and 2).

**Figure 3A and B.**
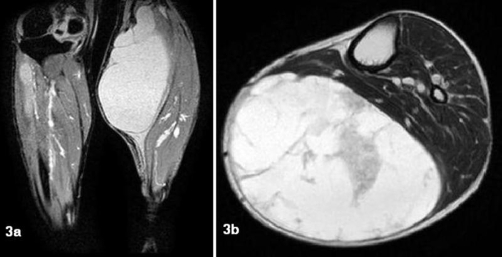
MRI showing a cystic lesion in the posteromedial aspect of the knee and calf.

Full blood count and inflammatory markers were normal. X-rays of the knee and tibia were normal. An ultrasound scan of the mass revealed the cystic nature of the swelling. A provisional diagnosis of a Baker’s cyst was made. An MRI of the knee and leg was obtained which revealed a large cystic lesion measuring 24 × 10 × 12 ;cm in the posteromedial aspect of the knee and calf. The lesion was reported to have multiple septae and was closely related to the semimembranosus muscle (Figures 3a and 3b). Following the MRI an excision was planned.

At surgery the swelling was found to be originating within the superficial posterior compartment of the leg without any communication with the knee joint or proximal tibio-fibular joint. The fluid inside the cyst was found to be mucinous. A definitive capsule was identified and the cyst was excised with its capsule.

**Figure 4.**
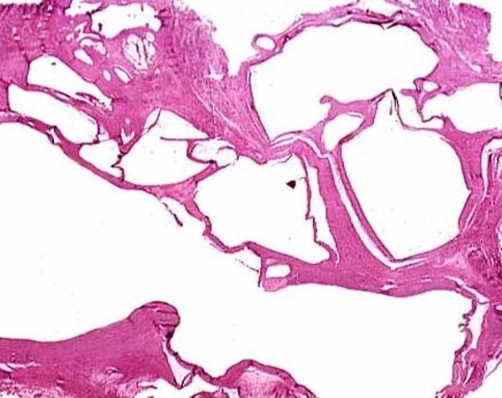
Histology of ganglion showing meshwork of fibrous tissue.

Histology revealed the mass to be a ganglion (Figure 4). The ganglion consists of a meshwork of fibrous tissue; the meshes of which are occupied by colloid material. There was no endothelial lining and the fibrous tissue appeared to have been derived by a process of degeneration from the surrounding tissue.

**Figures 5A and B.**
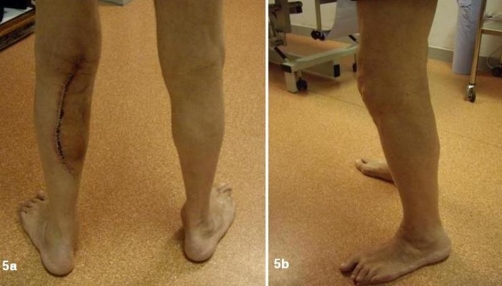
Postoperative images of patients leg after excision of the ganglion.

The patient had an uneventful recovery with complete resolution of all symptoms (Figures 5a and 5b).

## Discussion

A ganglion is a term applied to a cyst filled with colloid material which is met within the vicinity of a joint or tendon sheath. The commonest variety-the carpal ganglion-is met with a smooth, rounded, or oval swelling on the dorsal aspect of the carpus, usually towards its radial side. Ganglia are thought to arise from cystic degeneration in a tendon sheath or joint capsule usually in middle aged males [3]. Previously it has been postulated that popliteal cysts occur more commonly with increasing age and in males as there tends to be an increasing prevalence of intra-articular pathology in these groups of people [4].

Other theories for the development of popliteal cysts suggest that these cysts may be the result of a lack of support from surrounding structures which leads to a gradual disturbance in the mechanics of movement and the production of excess fluid in a synovial membrane [3]. A hernial protrusion of a synovial membrane of a joint or tendon sheath often occurs with the resultant formation of a ganglion. We have not been able to demonstrate any communication between the cavity of the cyst and that of an adjacent tendon sheath or joint. It is possible that this cyst may originate from a minute portion of synovial membrane being protruded and strangulated so that it becomes disconnected from that to which it originally belonged. It may then degenerate and give rise to colloid material, which accumulates and forms a cyst.

Large synovial cysts have been reported in patients with rheumatoid arthritis and a Baker’s cyst is a common finding in patients over 50 years old. However, it is very rare for a ganglion to reach a size of 24 × 10 × 12 ;cm and we have not found any case reports similar to this.

The differential diagnosis of ganglia in the calf and popliteal fossa can be demanding. Differentials such as a Baker’s cyst, haematomas, lipomas and a deep vein thrombosis must be excluded. Ultrasound can be used however is not adequate to visualise associated structures. MRI is the most accurate imaging modality and is best used for planning the surgical excision [5]. Although rare, ganglion cysts should be kept in mind while evaluating masses in the popliteal fossa and calf region. This case also highlights the importance of conducting accurate diagnostic tests in planning the surgical excision.

## Abbreviation

MRI: Magnetic Resonance Imaging
